# Positive experiences of a vocational rehabilitation intervention for individuals on long-term sick leave, the Dirigo project: a qualitative study

**DOI:** 10.1186/s12889-017-4804-8

**Published:** 2017-10-10

**Authors:** Åsa Andersén, Christian Ståhl, Ingrid Anderzén, Per Kristiansson, Kjerstin Larsson

**Affiliations:** 1 Department of Public Health and Caring Sciences, Sociomedical Epidemiological Section, Uppsala University, Box 564, SE-751 22 Uppsala, Sweden; 2 National Centre for Work and Rehabilitation, Department of Medical and Health Sciences, Linköping University, SE-581 83 Linköping, Sweden; 3 Department of Public Health and Caring Sciences, Family Medicine and Preventive Medicine Section, Uppsala University, Box 564, SE-751 22 Uppsala, Sweden

**Keywords:** Sick leave, Vocational rehabilitation, Motivational interviewing, Cooperation, Return-to-work, Qualitative study

## Abstract

**Background:**

The process of returning to work after long-term sick leave can sometimes be complex. Many factors, (e.g. cooperation between different authorities and the individual as well as individual factors such as health, emotional well-being and self-efficacy) may have an impact on an individual’s ability to work. The aim of this study was to investigate clients’ experiences with an individually tailored vocational rehabilitation, the Dirigo project, and encounters with professionals working on it. The Dirigo project was based on collaboration between rehabilitation authorities, individually tailored interventions and a motivational interviewing approach.

**Methods:**

A descriptive qualitative design was used with data collected through interviews. Fourteen individuals on long-term sick leave took part in individual semi-structured interviews. The interviews were analysed using content analysis.

**Results:**

The analysis showed overall positive experience of methods and encounters with professionals in a vocational rehabilitation project. The positive experiences were based on four key factors: 1. Opportunities for receiving various dimensions of support. 2. Good overall treatment by the professionals. 3. Satisfaction with the working methods of the project, and 4. Opportunities for personal development.

**Conclusions:**

The main result showed that the clients had an overall positive experience of a vocational rehabilitation project and encounters with professionals who used motivational interviewing as a communication method. The overall positive experience indicated that their interactions with the different professionals may have affected their self-efficacy in general and in relation to transition to work. The knowledge is essential for the professionals working in the area of vocational rehabilitation. However, vocational rehabilitation interventions also need a societal approach to be able to offer clients opportunities for job training and real jobs.

**Electronic supplementary material:**

The online version of this article (10.1186/s12889-017-4804-8) contains supplementary material, which is available to authorized users.

## Background

Long-term sick leave may lead to negative consequences such as impaired psychological well-being and sleep disturbances [1], feelings of powerlessness [2] or inactivity and isolation [1, 3]. People with long-term sickness absenteeism also have a high risk of a future disability pension [4]. In Sweden, mental illness and musculoskeletal diseases constitute the most common reasons for long-term sick leave (≥60 days) [5]. Longer durations of sick leave (≥60 days) increases the need for vocational rehabilitation [5], which are rehabilitation interventions aimed at facilitating return-to-work (RTW) [6].

The rehabilitation process for RTW can sometimes be complex and many factors can influence an individual’s work ability [7]. Factors linked to the individual (including physical, cognitive, affective and social domains) and elements in the environment, such as the workplace system (e.g., environment, organisation, work relations, work load), the healthcare system and the compensation system influence the disability, which must be taken into consideration by the authorities that partake in rehabilitation processes to facilitate RTW [8].

The Swedish Social Insurance Agency (SSIA) is in charge of coordinating the rehabilitation process and is the responsible actor for the administration of sickness benefits in Sweden. Other authorities involved in vocational rehabilitation with different responsibilities are (1) the health care provider, with responsibility for medical rehabilitation; (2) the Swedish Public Employment Service (SPES), with responsibility for vocational rehabilitation; (3) municipalities, with responsibility for social rehabilitation; and (4) the employers [9]. The client on sick leave is dependent on how these authorities work and the decisions they make [10]; and lack of cooperation and communication among them is an obstacle to the RTW process [11]. Since vocational rehabilitation programs should be planned in cooperation between the authorities and the clients and designed according to the needs of the client to reach a positive outcome [12], they may have various designs.

The relationship and cooperation between the client on sick leave and the authorities that work with vocational rehabilitation is important for the client’s RTW [12]. It has been argued that there is a need for more cooperation between various authorities to better meet the needs of clients on sick leave [13] and provide them with proper support [7]. However, research shows varying results regarding cooperation in vocational rehabilitation [14–17]. Some studies point to positive effects of collaboration [18, 19], one study failed to see any significant effects at all [20] and another study found negative effects of RTW [17]. Overall, however, the cooperation between authorities seems to be regarded as a positive idea that is being reviewed by both employees [20, 21] as well as clients [22, 23].

Furthermore, several researchers seem to agree that co-location of different organisations, or parts of organisations, into a common local area creates good conditions for cross-border cooperation [24] In a study, co-location of organisations is even described as a “full integration” [25]. In Sweden, several attempts have been made with co-location of state and municipal authorities in so-called *med-citizen* or service offices [26].

The principles of motivational interviewing (MI) have been used to improve RTW in a number of vocational rehabilitation interventions, including different populations with various conditions or illnesses, which is shown in a recently published review [27]. MI is a client-centered communication method aimed at facilitating behavioural changes by strengthening an individual’s motivation and commitment to change.

In MI, active collaboration between advisor and client is emphasised, as the client is believed to be the one who best describes his current state of health and the psychosocial situation [28]. MI is based on the principles of partnership, acceptance, compassion and evocation. The *partnership* should be based on cooperation between the professional and client, where clients should be seen as experts on themselves. *Acceptance* includes seeing the value of every human, striving to understand the client’s perspective, showing empathy, respecting the client’s autonomy and confirming the client’s strengths. *Compassion* is shown by actively trying to help clients feel good, with a focus on their needs. *Evocation* is about eliciting the client’s own motivation for change. Furthermore, another important foundation for clinical use of MI is active listening [28], i.e., the expression of empathy, the development of discrepancy and avoidance of argumentation, as well as the abilities to roll with resistance and support self-efficacy [29].

Two recently published studies found that RTW was both improved and more sustainable for workers with disabling musculoskeletal disorders when MI was added to a routine work rehabilitation intervention compared with controls who did not receive MI [30, 31].

The following study is part of an evaluation of a Swedish vocational rehabilitation project, the Dirigo project, targeting individuals on long-term sick leave. The Dirigo project has been described in detail in a previous publication focusing on organisational and professional aspects [32]. The project took place in two municipalities in the southern part of Stockholm from January 2012 to April 2014. The intervention of the project was directed to three groups: 1) clients on long-term sick leave (>180 days), 2) youth with disability benefits (benefits for long-term reduced working capacity in young adults 19–29 years), or 3) recipients of social allowances. In the present study, only Group 1 is included. The inclusion criteria were a diagnosis corresponding to mental illness (anxiety, mild depression, and stress problems) and pain-related problems. The exclusion criteria were suicidal risk, serious physical illness or injury based on the criteria of the Swedish National Board of Health and Welfare [33], another ongoing treatment, or participation in another cooperation project. Potential participants in Dirigo were identified by the SPES, SSIA or the municipalities and referred to the project. The Dirigo project developed unique features compared to regular practice and was built on the following three pillars: the direct collaboration between the SSIA, SPES and the municipalities, the individual tailored interventions and the motivational interviewing approach.

In the Dirigo project, the professionals shared workplaces in two dedicated offices, worked together in pairs, and shared responsibility for a case. This close cooperation between the authorities differs from regular practice where the professionals generally work alone and only collaborate with other authorities at specific time points [34].

The professionals also worked closely with the clients to support their individual vocational rehabilitation process. The professionals had relatively few cases; the caseload was about 30–40 per professional compared with over 100 in regular practice. This allowed the professional to spend more time with the clients and offered more flexibility in terms of where and when meetings with clients could be held. For example, professionals carried out several meetings in locations other than the office, such as on walks, and could accompany clients to various meetings within the framework of the vocational rehabilitation process.

All professionals working in Dirigo received MI-training at the beginning of the project and the principles of MI [28] were used as a guideline for meetings with clients. MI was used as a tool to improve both cooperation between clients and professionals, i.e., the principle of partnership, and to improve client and professional communication and alliance, i.e., the principles of acceptance and compassion. MI was also used as a means to strengthen clients’ motivation for transition to work, i.e., the principle of evocation.

The aim of this study was to investigate clients’ experiences with an individually tailored vocational rehabilitation, the Dirigo project, and encounters with professionals working in it.

## Methods

### Design

Because qualitative methods are suitable for studying thoughts and experiences and how they affect people [35], a descriptive qualitative design was chosen with data collected through interviews. The study was conducted alongside a vocational rehabilitation project, the Dirigo project, with the main aim of improving the clients’ work ability to support transition to work or commencement of studies.

### Selection and recruitments

Participants (clients) for this study were chosen purposively to attain diverse opinions and experiences with the project and encounters with the professionals. The clients included were selected from the vocational rehabilitation project and asked by the professionals if they wanted to participate in an interview. The professionals were told to ask both women and men of various ages to obtain variation in the sample.

### Data collection

A total of 14 face-to-face interviews were conducted with project clients, including eight women and six men, mean age 47.2 (range 27 and 59 years). Most of the clients had been on sick leave for 6 to 18 months. See Table 1 for an overview of the clients’ characteristics. The clients received verbal and written information about the purpose of the study and were informed that participation was voluntary. All clients consented to participate. The interviews were semi-structured, carried out between May and November 2013, and were conducted by the first (Å.A.) and last (K.L.) authors. The clients were asked about how they perceived the reception area in the project, their opinion about the activities offered by the project, their own goals with participation, needs for goal fulfillment and whether these were met by the project. Furthermore, questions about what they missed in the project, opinions about their opportunities to begin work or study and whether the project collaborated with their network was included in the interview guide (see Additional file 1: Interview Guide). The present study focuses on the clients’ experiences of the vocational rehabilitation project and their encounters with the professionals working in it. The interviews were 20–60 min long and were held at the project’s two sites. All interviews were audio-recorded and transcribed by an independent transcription service. Recruitment of respondent ended when data saturation was deemed to be reached.

**Table 1:** Overview of clients’ characteristics

	Gender	Age	Ethnicity	Reason for sick-leave (self-reported)	Time for sick-leave
1	Male	56	Immigrant^a^	Pain, depresion	15 months
2	Female	59	Native	Chronic obstructive pulmonary disease	18 months
3	Male	56	Native	Cancer	No information
4	Female	56	Native	Pain, depression	6 months
5	Female	28	Native	Depression	6 months
6	Male	36	Native	Depression, alcohol abuse	12 months
7	Female	45	Native	Bipolar disease	No information
8	Female	27	Native	Depression, borderline	6 months
9	Male	57	Native	Depression, bipolar disease, high blood pressure	12 months
10	Female	55	Native	No information	No information
11	Female	43	Native	Depression, pain	16 years
12	Male	58	Native	Depression, obesity	18 months
13	Female	42	Native	Depression	No information
14	Male	43	Native	No information	Over 5 years

^a^First generation immigrant

### Researchers’ background and ethical considerations

Four of the researchers involved in the interviews and analyses are not clinicians in vocational rehabilitation services. Of these researchers, one is a public health scientist, one is a social worker, one is a behaviourist, and one is a social scientist. The fifth researcher is a general practitioner engaged in rehabilitation processes of people with various diagnoses. The study was carried out according to the Code of Ethics of the Declaration of Helsinki and approved by the Regional Ethical Review Board of Linköping (Reg. no. 2012/115–31). Informed consent was obtained from all clients before participation.

### Data analysis

The qualitative analysis used inductive content analysis with a manifest (i.e., what is pronounced) approach [36]. The analyses were performed by the first (Å.A), second (C.S.) and last (K.L.) authors as follows: the interviews were read through to focus on the issues and get a sense of the entire text. In the next step, meaning units corresponding with the aim were identified, condensed and coded. These three researchers performed these initial steps independently. By classifying the codes as belonging to a specific group, they were sorted into different subcategories. Through abstraction of the content within the subcategories, they were grouped into generic categories and finally into one main category [36]. During the analysis process, discussions concerning the coding and categorisations were held among the analysts until consensus was reached.

## Results

**Fig. 1:**
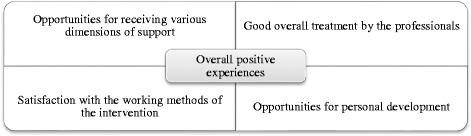
Illustration of the relationship between the overall experience and key factors, according to the clients

The main result is that the clients had overall positive experiences with the rehabilitation project and encounters with the professionals working in it. Their positive experiences are based on the following key factors: 1. Opportunities for receiving various dimensions of support. 2. Good overall treatment by the professionals. 3. Satisfaction with the working methods of the project, and 4. Opportunities for personal development. The relationship between the main result and the key factors is illustrated in Fig. 1.

### Opportunities for receiving various dimensions of support

#### Personal and emotional support

Clients were supported by the professionals to find balance in life, go outside their homes, set goals and make decisions. This support was perceived as valuable and as providing a sense of relief and the feeling that they are not undergoing the process alone.

*“… they [the professionals] are on my side in some ways”.* (no. 13).

#### Work-focused support

Mapping generated support and help from the professionals, as well as support in finding and maintaining employment or studies. The professionals were seen as coaching and followed up with the clients while they were at job training, which produced a sense of security regarding the new work or study circumstances. The clients felt the professionals wanted to help them return to work and said the professionals coordinated contact with various specialists, for example, vocational guidance counselors.

*“They [the professionals] were able to coach me a little, with finding work and such, so I think the support is good”.* (no. 7).

#### Procedural support

This support mainly involved the professionals helping to manage sickness benefits. The clients reported that the professionals offered information and answered various questions, which contributed to a decrease in their worries. They also received help from the professionals through assistance with filling in forms, requesting and seeking medical certificates, and making sure the sickness benefit case proceeded as it should, which clients appreciated and considered a relief.

*“… help with papers at the Social Insurance Agency and things like that; this was also practical, so I’ve had great support with all of this”.* (no. 7).

### Good overall treatment by the professionals

#### Overall treatment

Clients were satisfied with how they were treated by the professionals, which was characterised as honest and open. The clients felt that the professionals were nice and caring, that the professionals treated them well and were engaged in their recovery process. Clients felt they could be relaxed around the professionals and described having confidence in them. The professionals were perceived as keeping their agreements, which was appreciated. The clients also felt the professionals trusted them and that they were non-judgmental and non-accusatory; they also had an understanding of the clients’ circumstances and their diseases. This overall treatment gave the clients a sense of security and a sense of being cared for.

*“… it feels like being embraced a little bit; you can sit down and feel safe.”* (no. 5).

#### Being seen as individuals believed in and treated respectfully

The clients felt the professionals trusted them; they did not feel questioned and they did not have to defend themselves. The clients felt the professionals had an individual approach to their treatment. The professionals were described as being respectful, as taking the clients seriously and as taking the time to listen to them.

*“They [the professionals] have been flexible; they have listened to me seriously, and have not dismissed what I have said …”* (no. 3).

Despite the good treatment, one client described negative experiences in meetings with one of the professionals. This client did not feel understood, actually felt ill-treated, and found the professional to be incompetent. However, after this client contacted the project manager, a new contact was appointed, which worked out better.

### Satisfaction with the working methods of the project

#### High expectations for receiving help from the project

The first meeting with the professionals was perceived as unconditionally and resulted in high expectations for receiving help from the project. The fact that the professionals asked them what they needed help with strengthened these expectations. The clients trusted the professionals and hoped their participation in the project would lead to something positive for them. The project was perceived as helpful by all clients and was perceived by the professionals as being so promising that the clients wished the authorities would adopt this working method broadly in the future.

*“I say, I have never been cared for like this, so I’m keeping my fingers crossed that this will be good”.* (no. 12).

#### Cooperation between the participant and the authorities

The clients appreciated the close cooperation between themselves and the authorities, which produced the sense of being part of a team. According to the clients, working together gave the professionals an understanding and an overview of the individual’s situation. This working method also gave the clients a sense of confidence, because it improved communication, providing them with answers irrespective of which authority they asked questions.

*“…regardless of who [among the professionals] I’ve turned to; I’ve gotten an answer”…* (no. 8).

The cooperation among the various authorities (the SSIA, SPES and the municipality) was appreciated by the clients and perceived as giving the professionals a better understanding of each other’s working methods. This cooperation was also seen as reducing the risk of the clients falling in between the authorities’ responsibilities. The clients thought this cooperation increased the authorities’ capacity compared with traditional approaches to vocational rehabilitation.

[Before the project] *“…the Public Employment Services sent me here and there, so it was such a juggling act with patients going from health care to the Public Employment Services, back and forth. Avoiding that and having two authorities working together is great; it couldn’t be better”.* (no. 10).

#### Individually designed working method

The working method was described as individually designed according to clients’ wishes, needs and conditions. The clients felt the professionals were there for them and took their needs into account, i.e. they adjusted the pace to suit the individual, and clients saw this way of working as unique. The professionals were perceived as making an effort to get to know the clients; they had personal conversations with them over time. As a result, the professionals could observe the individuals’ resources, needs and what occupations suited them.

*“… they [the professionals] have not pushed me to do things, nor have they let me do nothing. There has been a balance and they have ensured that everything starts from me”.* (no. 8).

#### Accessible, continuous and frequent contact

The clients found the professionals to be accessible and easy to keep in touch with by e-mail or phone. Clients appreciated having continuous and frequent contact with the same professionals over time, which produced a sense of security and made them feel like the professionals were there for them. The professionals were also perceived as having more time for each participant compared with regular practice. However, the clients would have liked more time with the professionals.

*“There is a little bit more time here for working with…clients in a stronger way…and a better opportunity for returning to work”.* (no. 14).

#### The professionals provided useful tools throughout the project

Among these tools were group activities organised within the project, which were considered important because they provided opportunities to experience new activities. The clients stated that these tools established contact with them and supported their vocational rehabilitation process.

*“… the program itself has helped them [the professionals]as a tool to better reach me and maybe to better help me”.* (no. 14).

### The project provided opportunities for personal development

#### Increased self-awareness in and belief in one’s own ability

Increased self-awareness included changes in self-perception and the skill of learning to listen to themselves. Clients said they got to know themselves better and to discover and become aware of the available opportunities for work or studies. By taking part in mapping (i.e., creating an overview of clients’ background, education, competence and goals or wishes regarding future work or studies), some clients reached a clearer understanding of themselves and what they wanted to work with, which gave them hope for getting a job. The project was seen as giving them a chance to look for different kinds of jobs, as well as the possibility to test their work capacity via job training. Clients also stated that taking part in the project increased their belief in the possibility of starting to work. Furthermore, clients reported seeing their self-worth and gaining insight into their own priorities.

*“… yes, but I would like to do it, but I didn’t think it was possible [working with a particular profession], and they [the professionals] made me believe it was possible”…* (no. 8).

#### Social contacts

For some clients, participation in the project led to the acquisition of new social contacts*.* Clients also talked about the joy of getting to know new people.

Through contact with the professionals, clients also discovered that there were others in similar situations, which produced a sense of ease and confirmed that they were not alone in their situation.

*“…when they [the professionals] said they knew others [clients] who felt bad, then I thought, God, what a relief, that they had met others”…* (no. 13).

## Discussion

The main result of the qualitative analysis was the consensus that the clients had overall positive experiences with the rehabilitation project and encounters with the professionals working in it. The positive experiences were based on the following key factors: the intervention provided opportunities for receiving various dimensions of support, good overall treatment by the professionals, satisfaction with the working methods of the project, and opportunities for personal development.

The clients felt that they received support, that they were cared for and treated with respect by the intervention professionals. Moreover, the clients experienced that the intervention, especially the cooperation between the authorities, allowed the professionals to spend more time with each individual compared with regular practice. The professionals were also perceived as having high availability and continuity in their contacts with project clients, which facilitated an appropriately individualised rehabilitation plan.

The clients’ experiences of their contacts and interactions with the authorities and the professionals working in the project are in line with previous research indicating that a cooperative approach resulting in a coordinated and tailored rehabilitation plan is considered positive and helpful by the individual [23]. This was described in our study in strong contrast to past experiences of the regular treatment by authorities. Although cooperative approaches vary in terms of intervention design, participating actors, target group, and expected outcome, there is a common understanding that a coordinated rehabilitation process is perceived to be helpful for the individual [34]; our results reinforce this.

Comparing results from studies involving cooperation in individually tailored rehabilitation is difficult because the context, design, and sample usually differ. It is also difficult to accurately measure the effects of cooperation, because it is often not possible to determine whether the observed effects depend on the cooperation itself, on other aspects of the intervention, or on a combination of all of these factors [37]. In our study, the cooperation between professionals was a central aspect of the intervention, but this was combined with the use of MI [29] as a tool for meeting the participants.

### Opportunities for receiving various dimensions of support

The clients felt that they received both emotional and instrumental support in terms of having someone on their side, helping them set goals, and receiving coaching and guidance. The clients in Dirigo claimed that the intervention had strengthened their beliefs in their abilities, which may be related to previous findings that support from professionals and family during vocational rehabilitation facilitated RTW for patients with musculoskeletal and/or psychological disorder [38]. Emotional support from professionals that is perceived as having someone to stand up for them is considered an essential part of a positive encounter between sick-listed persons and rehabilitation professionals [39]. There may also be a need for support from professionals to move forward in the rehabilitation process. This support may include e.g., assistance with making contacts, job training arrangements and follow-up meetings [40]. In the Dirigo project, the clients received help with contacting employers and support during job training from professionals. This may be seen as a stepwise transition to work in parallel with consultations with professionals that may have helped clients to overcome various barriers in transition to work.

### Good overall treatment by the professionals

The clients expressed how their encounters with the professionals were perceived as empowering and respectful, and that the good treatment brought them a sense of confidence in contacts with the professionals. This may be related to previous studies on the importance of fair treatment, and that the quality of encounters has an important influence on the self-perception of the clients, including their expectations of being able to work [41, 42]. Previous research has shown that the clients’ sense of confidence during vocational rehabilitation is important when communicating important information to the professionals so that they can correctly assess the client’s work ability and appreciate their difficulties and resources [43]. The manner in which the clients describe the overall treatment by the professionals reflects the fundamentals of MI, i.e., cooperation and partnership, showing respect toward the client, confirming the right to self-determination (autonomy) and active listening [28].

When one of the clients was dissatisfied with a contact and complained to the project management, the complaints were resolved immediately by appointing a new contact. This measure may be in line with the project’s readiness to show participants respect and trust.

### Satisfaction with the working methods of the project

The participants expressed positive attitudes toward the project and the working methods, which were considered helpful. The professionals were perceived as highly available and as having continuous contact with participants, which was seen as helpful to the development of an appropriate and individualised rehabilitation plan. Previous research has shown that a cooperative approach resulting in a coordinated and tailored rehabilitation plan is considered positive and helpful by individuals on sick leave or who are out of work due to common mental disorders [22]. Participating in decisions regarding one’s own vocational rehabilitation has also been shown to be an important factor for the individual’s RTW [39]. In our study, the close cooperation between the participants and professionals involved the participants in the rehabilitation process. If there is a structured plan for the vocational rehabilitation process and the individual on sick leave understands what will happen, what steps will be taken and who is doing what, this could support the RTW process [40].

In the Dirigo project, professionals used the principles of MI when communicating with clients. MI has been considered to be a key to successful RTW in studies in other countries [30, 31]. One of the components in MI is to support self-efficacy [29], which has been shown to be important in promoting health and the return to work [42]. A previous study showed that when professionals worked according to MI, it increased self-efficacy in individuals by supporting their belief that they could accomplish the actions needed to reach their goals [44]. The association between MI and increased self-efficacy has been shown in previous studies in areas such as physical activity and self-management strategies [45, 46]. Using MI may be considered to be a step in offering relevant social support (e.g., through regular contact with the professionals and group activities), and has also been associated with higher self-efficacy [47–49]. The clients in the Dirigo project expressed that they had increased their self-awareness during the intervention, which may have had an impact on their future opportunities to return to work. Hence, the results of this study are likely to have been influenced by a combination of the use of MI, the close cooperation between professionals, and the organisational conditions allowing the professionals to have continuous and close contact with the clients.

### Opportunities for personal development

The clients experienced, on a personal level, increased self-awareness and belief in their own ability, i.e., increased self-efficacy, and that they had gained new social contacts through participation in the intervention.

They expressed that their encounters with the professionals strengthened their self-awareness, self-worth and belief in their ability to start working or studying. In a previous study, self-awareness, such as identity, resources, will and values were found to be facilitators of returning to work after sick leave [38]. The interactions that may occur between the professionals and individuals involved in vocational rehabilitation have also been found to have an important influence on the individuals’ perceived abilities, including their expectations about or belief in being able to work [41].

The result in this study shows that the interactions between professionals and clients induce most positive outcomes, depending on the competence of professionals and how clients experience the encounters. The answers in the interview with the clients indicate that their interactions with the different professionals may affected their self-efficacy, and thus, possibly the outcome of the received intervention and the transition to work. Whether or not our study participants began to work is not the focus of the present study. However, these approaches were primarily individual and for a successful transition to work, vocational rehabilitation interventions need a further societal approach to actually be able to offer clients opportunities for job training and real jobs.

### Methodological considerations

Since the aim of this study was to capture client’s experiences, a qualitative design with content analysis was chosen. Content analysis is a method that has been used for a long time in qualitative studies, for example in the area of public health. This data analysis method was considered appropriate since it can be used to analyse verbal and written communication [36]. A purposive sampling method was used to reach both women and men of various ages to capture different experiences with the research questions. The sample size was judged to be sufficient when saturation in the collected data was considered to be reached. To strengthen credibility, the first steps of the analysis were performed independently by the three researchers, followed by discussions in the research group, until the codes and categories were consistent [50]. Dependability was strengthened by transparently describing the steps of the research process [50]. To achieve confirmability, authentic citations were presented, illustrating the data from which the categories were formulated [51].

There are also limitations worth noting. The participants in the interviews were recruited by professionals with whom they already had a relationship. The professionals were asked to include clients who were willing to talk about their experiences of the project, both women and men and of various ages. However, our sample was older (mean age 47.2) and included fewer women (57%) than the Dirigo project (mean age 42.5 years, 64% women). The interviews were performed at the project site, which could have affected the outcome. Furthermore, it is unclear how many individuals declined to participate in our study and how they experienced the project and encounters with the professionals. With this in mind and the fact that this is a qualitative study which does not claim generalisability, the results should be interpreted with caution and is primarily valid for these clients.

## Conclusions

The clients had overall positive experiences of the vocational rehabilitation project Dirigo and encounters with the professionals working in it. Their positive experiences were based on four key factors: 1. Opportunities for receiving various dimensions of support. 2. Good overall treatment by the professionals. 3. Satisfaction with the working methods of the project, and 4. Opportunities for personal development.

The professionals used the principles of MI when communicating with the clients. MI has been considered to be a key to successful RTW in other studies. The answers in the interview with the clients indicate that their interactions with the different professionals may have affected their self-efficacy in general and in relation to transition to work. Self-efficacy has shown to be a predictor of RTW after long-term sick leave in previous research. The knowledge is essential for the professionals working in the area of vocational rehabilitation. However, vocational rehabilitation interventions needs a societal approach to be able to offer clients opportunities for job training and real jobs.

## Additional file

Additional file 1: Interview Guide. This document contains the semi-structured questions used in the interviews (DOCX 19 kb)

## Abbreviations

ESF: European Social Fund
MI: Motivational Interviewing
RTW: Return to work
SPES: Swedish Public Employment Service
SSIA: Swedish Social Insurance Agency
