# Supplementary material for: Positive experiences of a vocational rehabilitation intervention for individuals on long-term sick leave, the Dirigo project: a qualitative study
Source: BMC Public Health. 2017 Oct 10;17:790. doi: 10.1186/s12889-017-4804-8 (PMC5633870; doi:10.1186/s12889-017-4804-8)
Supplement: Additional file 1: — Interview Guide. This document contains the semi-structured questions used in the interviews (DOCX 19 kb) [file 12889_2017_4804_MOESM1_ESM.docx]

#####

#####

##### **Interview guide - participants**

How has the reception in the project been?

- Information
- Treatment
- Availability

What do you think about the activities offered in Dirigo?

e.g.

- Individual planning
- Team meeting
- Wellness activities
- Guidance for study
- Job training

What were your goals in participating in the project?

What do you need for these goals to be achieved?

Does the project meet the needs you have for achieving your goals?

What do you think is missing in Dirigo?

What do you think about your opportunities to start work or study?

What does your other network look like – is there a network-and-Dirigo collaboration?

What do you want to convey that I have not asked about?
